# Sunitinib Induced Thrombotic Thrombocytopenic Purpura in addition to Severe Hypothyroidism: A Case Report and Review of the Literature

**DOI:** 10.1155/2014/958414

**Published:** 2014-10-07

**Authors:** Imane El Dika, Deborah Mukherji, Sally Temraz, Rita Assi, Ali Shamseddine

**Affiliations:** Department of Internal Medicine, Division of Medical Oncology, American University of Beirut Medical Center, P.O. Box 11-0236, Riad El-Solh, Beirut 1107 2020, Lebanon

## Abstract

*Introduction*. Sunitinib malate is an oral multitargeting tyrosine kinase inhibitor approved for the first line treatment of metastatic renal cell carcinoma. Sunitinib administration is associated with several adverse events including fatigue, diarrhea, skin toxicity, hypothyroidism, and cytopenia. Herein, we present a case of thrombotic thrombocytopenic purpura and clinical hypothyroidism presenting within 4 weeks of starting sunitinib therapy. *Case Presentation*. A 72-year-old woman with metastatic renal cell carcinoma presented with generalized fatigue 28 days after starting sunitinib 50 mg daily. She was found to have severe hypothyroidism, in addition to significant thrombocytopenia and anemia. The latter were explained by a clinical and laboratory diagnosis of thrombotic thrombocytopenic purpura. Sunitinib was stopped and she recovered completely after plasmapheresis. *Conclusion*. To our knowledge, this is the fourth case report of thrombotic thrombocytopenic purpura secondary to sunitinib. Oncologists should be aware of this rare but potentially fatal adverse event. We highly suggest to routinely test for platelet count and thyroid stimulating hormone level as early as two weeks after initiating sunitinib.

## 1. Introduction

Sunitinib is an oral multitargeting tyrosine kinaseinhibitor that inhibits members of the receptor tyrosine kinases (RTK) families containing a split-kinase domain. These families include VEGF receptor (VEGFR) types 1 (FLT1), 2 (KDR), and 3 (FLT4); platelet-derived growth factor receptors A and B (PDGFRA and PDGFRB); the stem cell factor receptor (cKIT); FMS-like tyrosine kinase 3 (FLT3); colony-stimulating factor 1 receptor (CSF-1R); and glial cell line-derived neurotrophic factor receptor [rearranged during transfection (RET)]. It is clinically approved in the treatment of pancreatic neuroendocrine tumor [1], metastatic renal cell carcinoma (mRCC) [2], and imatinib resistant metastatic gastrointestinal stromal tumor [3]. However, the broad-spectrum activity associated with this multitargeted drug confers it with a unique toxicity profile. Reported adverse events include asthenia, diarrhea, skin toxicities, hypothyroidism, cardiotoxicity, hypertension, and myelosuppression [4]. Bleeding events and tumour hemorrhages have been reported in many patients receiving sunitinib for mRCC. It ranges from epistaxis to severe life-threatening hemoptysis or pulmonary hemorrhage. The inhibitory concentration (IC_50_) of sunitinib is 143 for PDGFR-alpha, 75 for PDGFR-beta, 21 and 34 for VEGFR 1 and VEGFR 2, respectively [5], which is higher than IC_50_ for other tyrosine kinase inhibitors, making sunitinib one of the most potent inhibitors of VEGFR and PDGFR.

Interestingly, fibroblast growth factor receptors, PDGFRs, VEGFRs, and their ligands have been shown to play important roles in tumor growth and angiogenesis. Inhibition of VEGF signaling through the use of antibodies or VEGFR antagonists has demonstrated the effect against tumor progression and metastasis.

Here we present the case report of a patient with metastatic renal cell carcinoma (mRCC) who developed thrombotic thrombocytopenic purpura (TTP) on sunitinib that was completely reversible after treatment withdrawal and plasma exchange therapy.

## 2. Case Presentation 

A 73-year-old female patient, with diagnosis of mRCC, presented with epistaxis and fatigue 28 days after being started on sunitinib 50 mg daily. Her medical history is significant for right radical nephrectomy in July 2009 for clear cell carcinoma, Fuhrman grade 4/4. In March 2012 she had left partial nephrectomy for recurrent disease. Pathology revealed clear cell carcinoma, Fuhrman grade 3/4, with negative surgical margins. In May 2013, she presented again with recurrence in the left kidney along with new right lung metastasis.

On clinical examination, she was pale and had diffuse ecchymosis with severe lower extremity edema. She had also decreased air entry at the lung bases. There were no fever and no neurological symptoms; she had no skin changes over her hands or feet and no diarrhea.

Relevant laboratory findings include a thyroid stimulating hormone (TSH) level of more than 100 *μ*U/mL (0.27–4.20 *μ*U/mL), hemoglobin of 8.8 g/dL (12–16 g/dL), and platelets count of 38000/mm^3^ (150000–400000/mm^3^). Creatinine increased to 3.2 mg/dL from baseline of 1.5 mg/dL (0.4–1.0 mg/dL) and lactate dehydrogenase (LDH) was 792 IU/L (110–265 IU/L). Her platelets count before initiating sunitinib therapy was 301000/mm^3^. Her hemoglobin was 11 g/dL. TSH level was 2.3 *μ*U/mL.

Her symptoms and cytopenia were initially thought to be due to severe hypothyroidism secondary to Sunitinib.

Sunitinib was then stopped and she was initiated on thyroid replacement hormone with supportive measures. During her hospitalization, she had systolic blood pressure readings reaching 170 mmHg, and her creatinine and proteinuria were not improving. Her platelet count reached a nadir of 12000/mm^3^. A blood film inspection interestingly revealed marked schistocytes; haptoglobin was 0.08 g/L (0.30–2.0); and reticulocyte count was 3.4% (0.2–2.0). Coagulation studies were normal and bone marrow evaluation was negative for malignancy.

**Figure 1:**
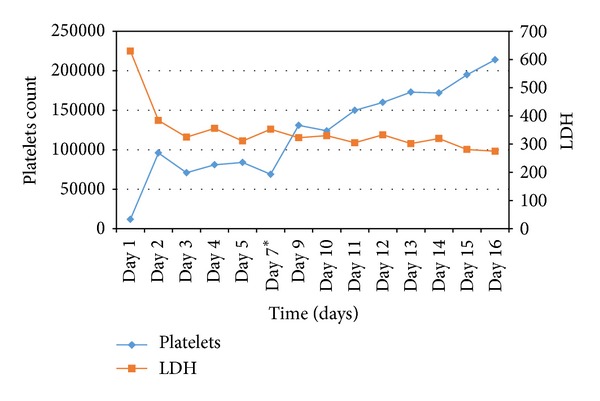
Platelets count and LDH values after each plasmapheresis session. *No plasmapheresis was done on Day 6.

In view of the above picture, the diagnosis of TTP was made. Plasma exchange was then started. After six sessions her blood counts values returned to normal, and creatinine level as well as TSH normalized. Platelet count and LDH level in response to plasma exchange sessions are shown in Figure 1. Sunitinib was therefore stopped definitely and the patient was advised to undergo tumor resection along with lung metastasectomy. However, she opted for best supportive care.

## 3. Discussion

Sunitinib is a tyrosine kinase inhibitor that targets VEGFR 2 and PDGFR-beta, which are both the major subtypes of VEGFR and PDGFR in capillary vasculature. It is hypothesized that sunitinib acts via direct anti-VEGFR and anti-PDGFR effects that result in damage of the capillary endothelium.

A number of chemotherapeutic agents have been proved to be associated with TTP-HUS, including gemcitabine, cisplatin, doxorubicin, oxaliplatin, and mitomycin C. Few cases of TMA or TTP-HUS were reported after sunitinib treatment. The pathophysiology of TTP-HUS and renal thrombotic microangiopathy (TMA) due to sunitinib is not very well understood.

Renal toxicity has been widely reported with bevacizumab, a VEGF pathway inhibition.

Hohenstein et al. [6] demonstrated in a murine model of site-specific (kidney) endothelial injury that VEGF was lost in many severely injured tubules and glomeruli (podocytes), but in parallel strongly upregulated in less severely injured tubules and glomeruli. This was associated with high platelet influx and presence of schistocytes in peripheral blood.

Frangié et al. [7] reported a case of TTP-HUS after treatment with anti-VEGF antibody bevacizumab for mRCC that recurred after use of sunitinib and required plasmapheresis. TMA and mesangiolysis were proven by renal biopsy.

Eremina et al. reported six cases for patients who developed glomerular injury in favor of TMA on treatment with bevacizumab [8]. Eremina et al. demonstrated that local reduction of VEGF within the murine kidney resulted in profound effects on the adjacent glomerular endothelium, causing significant damage to the fenestrated endothelium and microvascular injury, leading to thrombotic microangiopathy. VEGF production by podocytes seems to be required for normal functioning and maintenance of the adjacent glomerular endothelium.

Ansari and George reported a case of sunitinib induced immune-mediated thrombocytopenia [9] that was completely reversible after withdrawal of the drug and treatment by steroids and immunoglobulins. Chemotherapeutic and immunosuppressive agents typically cause thrombocytopenia by suppressing hematopoiesis. However immune-mediated thrombocytopenia is very well reported. Sunitinib has been associated with other autoimmune disorders such as hypothyroidism; therefore, the possibility of an immunologic phenomenon to account for sunitinib-induced immune-mediated thrombocytopenia is quite reasonable.

Talebi et al. reported a case of sunitinib induced microangiopathic hemolytic anemia (MAHA) with a fatal outcome despite treatment with plasmapheresis, dialysis, and withdrawal of sunitinib [10]. ADAMTS-13 inhibitor assay was negative.

Costero et al. reported the first case of histologically confirmed focal segmental glomerulosclerosis in addition to TMA secondary to sunitinib treatment [11].

Kapiteijn et al. reported a case of TMA and reversible posterior leukoencephalopathy syndrome (RPLS) due to sunitinib in imatinib resistant GIST [12].

Another case of TMA after treatment with sunitinib for refractory malignant skin hidradenoma was also reported by Bollée et al. [13]. He demonstrated histological renal involvement and hypothesized that all anti-VEGF drugs may share a common risk for developing renal adverse events. In contrast to bevacizumab induced TMA, complement alternative pathway was not involved. There were glomerular subendothelial deposits of IgA and IgM, similar to bevacizumab-associated deposits.

Choi et al. [14] reported a case of TTP-HUS secondary to sunitinib for mRCC.

Table 1 summarizes the reported cases of TTP secondary to sunitinib in the literature.

In contrast to chemotherapy induced TTP-HUS, sunitinib induced TTP-HUS does not seem to be dose dependent and has a better outcome after stopping the drug and plasma exchange therapy [15]. Most sunitinib-associated adverse events are related to its mechanism of action. Although molecular basis behind each toxicity is not very well understood, inhibition of VEGF signaling seems to be behind proteinuria, hypertension, and hypothyroidism.

## 4. Conclusion 

To our knowledge this is the first case report on simultaneous occurrence of TTP and severe hypothyroidism within 1 month of sunitinib treatment. This raises the hypothesis to look for early clinical signs of hypothyroidism, as early as 2 weeks after initiating therapy. Oncologists should instruct their patients about alarming symptoms and have high clinical suspicion for such rare adverse events. We highly suggest closer monitoring of TSH, kidney function, blood pressure, and platelet count in all patients treated with sunitinib or anti-VEGF therapy.

## Figures and Tables

**Table 1:** Summary of cases of TTP associated with sunitinib in the literature.

Author	Age/sex	Cancer	Dose/time of onset	Presentation	Treatment	Outcome
Kapiteijn et al. [12]	54 years/female	GIST∗	50 mg/day 6th cycle	Loss of vision, seizure, hypertension, and RPLS^§^	Withdrawal and plasma exchange	Recovery

Frangié et al. [7]	70 years/male	RCC^†^	ND^‡^/3 weeks	Asthenia, hypertension, and renal insufficiency	Withdrawal and plasma exchange	Recovery

Choi et al. [14]	62 years/female	RCC	50 mg/day 3 weeks	Hematuria, proteinuria, and hypertension	Withdrawal and plasma exchange	Recovery

This case	73 years/female	RCC	50 mg/day 4 weeks	Asthenia, edema, renal insufficiency, and anemia	Withdrawal and plasma exchange	Recovery

*Gastrointestinal stromal tumor.

^§^Rapidly progressing posterior leucoencephalopathy.

^†^Renal cell carcinoma.

^‡^Not described.
